# Supplementary material for: The Autophagy Nucleation Factor ATG9 Forms Nanoclusters with the HIV-1 Receptor DC-SIGN and Regulates Early Antiviral Autophagy in Human Dendritic Cells
Source: Int J Mol Sci. 2023 May 19;24(10):9008. doi: 10.3390/ijms24109008 (PMC10219569; doi:10.3390/ijms24109008)

SUPPLEMENTAL DATA

Figure S1. Relates to Figure 2.

Lysates from MoDC pre-treated 1h with bafilomycin A1 (50 nM) before incubation for 1h with DC-SIGN mAbs were immunoblotted with anti-LC3 (upper blot) and loading was controlled with anti-actin (lower blot). Densitometry values and LC3-II/actin ratio were graphically reported (n=3). Statistical significance: ns = not significant; * = $p < 0.05$; ** = $p < 0.01$; *** = $p < 0.005$.

Figure S2. Relates to Figure 4.

(A) Primary MoDC were analyzed as in Figure 4 upon treatment with DC-SIGN mAbs (left imaging panels). In parallel, lysates from MoDC untreated or treated or with DC-SIGN mAbs for indicated times were immunoblotted with anti-LC3 and anti-P62/SQSTM-1. Loading of samples was controlled with anti-actin immunoblotting. Immunoblotting experiments shown are representative of 4. **(B)** The same experiment as above was performed but with cells previously treated with torin (2 µM) and chloroquine (50 µM). Immunoblotting experiments shown are representative of 4. The white bar in immunofluorescence panels corresponds to 5 µm. Arrows denote areas of DC-SIGN and ATG9 co-localization. Arrowheads mark larger DC-SIGN$^+$/ATG9$^+$ vesicular structures.

Figure S3. Relates to Figures 2 and 3.

Ectopic DC-SIGN stably expressed in HEK293T cells induces autophagy flux and associates with ATG9. **(A)** HEK293T cells were transduced with DC-SIGN-expressing lentiviral vectors and cultured under puromycin selection to give rise to HEK-DC-SIGN cells. DC-SIGN expression was analyzed by flow cytometry for cell surface (left histogram) or intracellular (right histogram) levels. Untransduced parental HEK293T cells were used as control (in gray). (**B**) Lysates from HEK-DC-SIGN cells pre-treated for 1h with Bafilomycin A1 (50 nM) before stimulation with DC-SIGN mAbs as indicated were immunoblotted with anti-LC3. Loading control was done with anti-GAPDH. Ratio of densitometry values for LC3-II and GAPDH bands are indicated. The experiments shown is representative of three 1
(n=3). (**C**) HEK-DC-SIGN cells were transfected with plasmids control (ø) or encoding HA-tagged ATG9 WT or mutant (N99D) proteins as indicated. Thirty-six hours post-transfection, cells were stimulated with ManLAM (2 µg/ml) for 30 minutes before lysis. Lysates were then subjected to immunoprecipitation with anti-HA-coated agarose beads. Lysates inputs and washed immunprecipitates were then immunoblotted with antibodies as indicated below each blot. Input control was done with anti-GAPDH. The experiment shown is representative of three (n=3).

Figure S4. Relates to Figure 6.

Primary MoDC were treated with irrelevant siRNA (siCtrl) or siRNA against ATG9 (siATG9) and ATG9 expression was controlled by RT-qPCR (left graph). Lysates from MoDC transfected as above and pre-treated with bafilomycin A1 (50 nM) for 1h before stimulation with ManLAM (2 µg/ml) for 2h were immunoblotted with anti-LC3 (upper blot). Loading control was done with anti-actin (lower blot). LC3-II/actin ratio from densitometry analyses obtained from 3 independent experiments (n=3) were normalized to untreated controls of each siRNA condition and graphically represented (right graph).

Figure S1

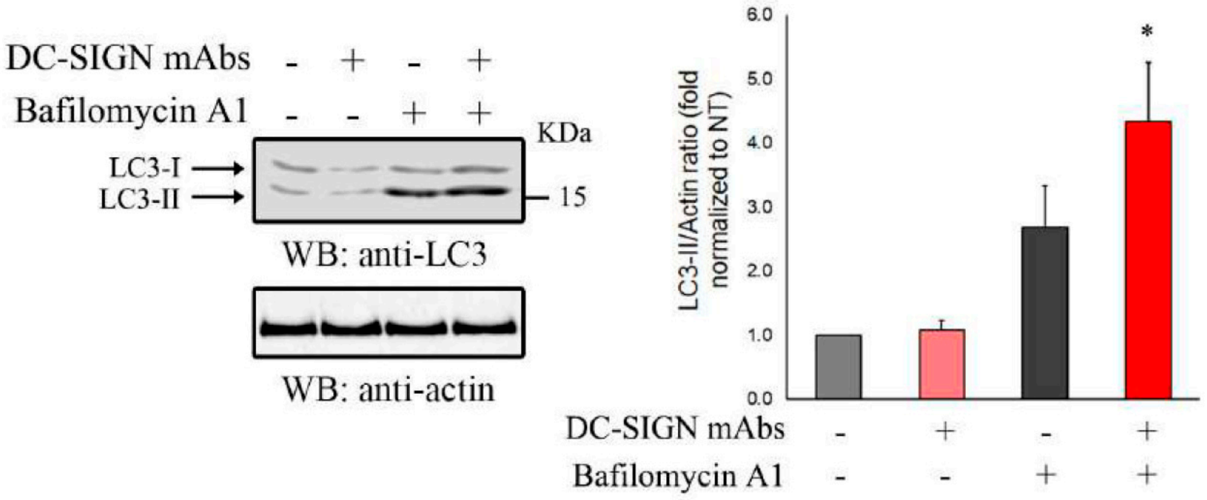

Figure S2

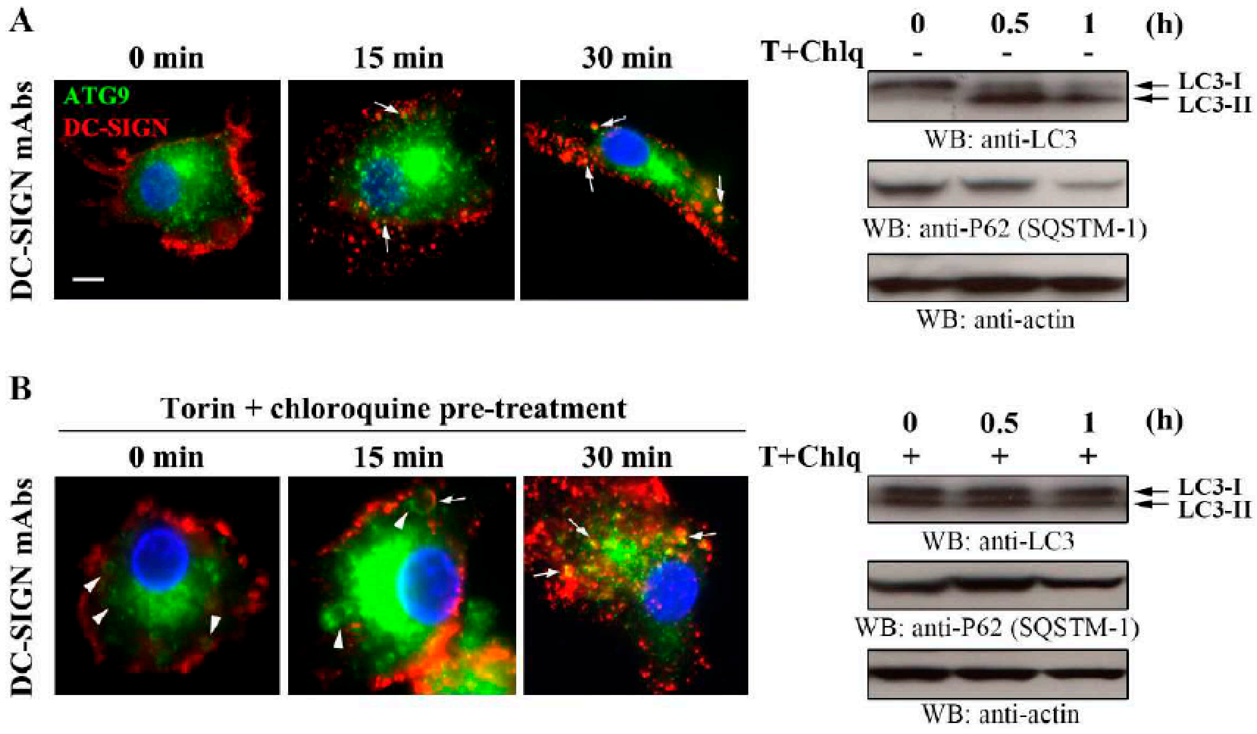

Figure S3

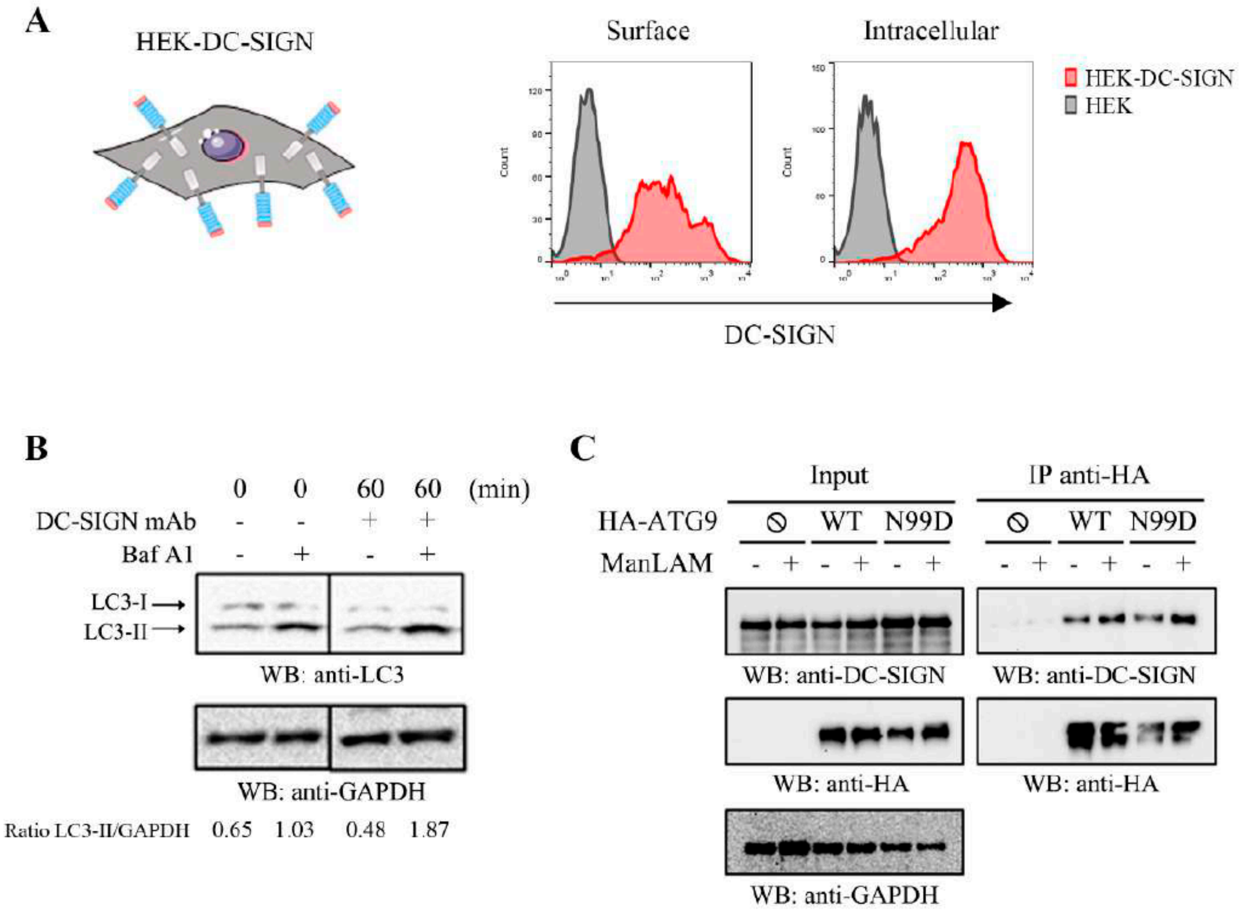

Figure S4

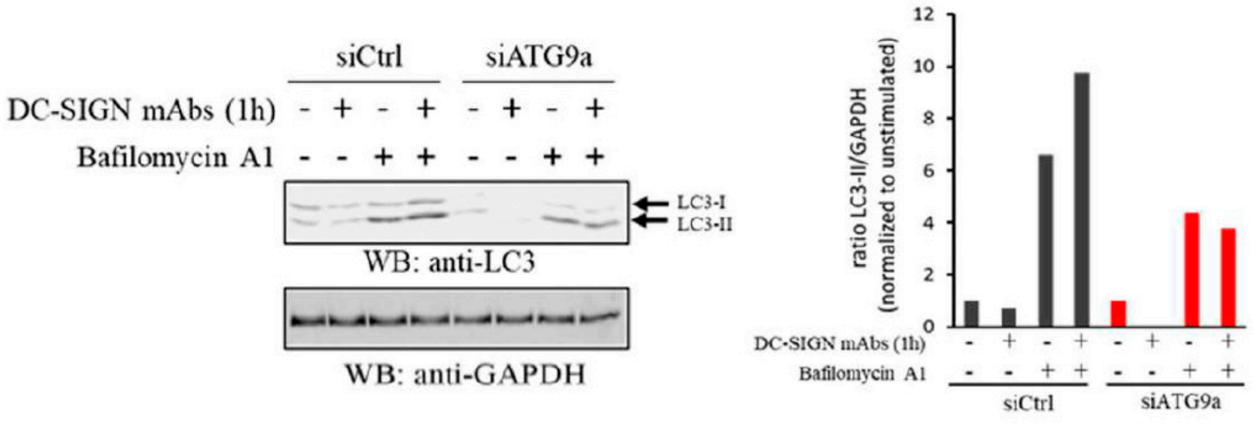

Supplement: Supplementary file 1 [file ijms-24-09008-s001.zip › ijms-2372553-supplementary.pdf]
